# Supplementary material for: Lung deflation while placing a subclavian vein catheter: Our experience in minimizing the risk of pneumothorax
Source: PLoS One. 2023 Feb 2;18(2):e0281300. doi: 10.1371/journal.pone.0281300 (PMC9894405; doi:10.1371/journal.pone.0281300)
Supplement: Attachment — Submitted filename: Response to Reviewers.docx [file pone.0281300.s001.docx]

Jamie Males

Editorial Office

PLOS ONE

We are very grateful to you for providing us with the opportunity to submit a revised draft of our manuscript titled, “Title: Lung Deflation while Placing a Subclavian Vein Catheter: Our Experience in Minimizing the Risk of Pneumothorax” to Plos One. We are highly appreciative of the time and effort that have been dedicated to providing us with your valuable feedback on the manuscript. We are also thankful to the reviewers for their insightful comments. We have been able to incorporate changes to reflect most of the suggestions provided and have highlighted the changes accordingly in the manuscript.

Enclosed herewith is a point-by-point response to the reviewers’ comments and concerns.

| Comments | Reply |
| --- | --- |
| Reviewer #1: | |
| An interesting manuscript in its field, I have no comments. This manuscript contains an interesting subject on the clinical field that any clinical doctor might encounter in the everyday clinical practice. | We are very grateful. The prospects of improving patient’s quality of care depend on repeated assessments of the measures taken. Therefore, this motivates us to explore unproven methods and validate the necessity to always question way to improve patients safety and outcome of care |
| Reviewer #2 |  |
| Overall, I think this is an interesting idea and potentially something to consider when placing SC lines. My main concern is that I am not convinced the measurements of the diameter were done accurately and consistently enough, and thus I am not sure of the validity of the results. I think it would be challenging for anyone to consistently measure the diameter of the vein in the same spot every time. I think a better description of how exactly these measurements were done and much better pictures would go a long way towards alleviating this concern. Additional specific comments and suggestions are given below. | Thank you very much for such an insightful comment.  We do agree about the limitations caused due to the possible variability in measuring the diameter of the subclavian vein. To minimize the chance of error, We have chosen to have one operator for the whole patient group as the probe pressure and placement might differ from one person to the other. As enclosed in the supporting file attached in the submission, We have addressed the issue by showing one of the initial steps to defining the diameter used in our database which would be taking multiple measurements and choosing the greatest diameter. We believe that a single measurement used can account for the variability in ultrasound probe pressure compared to calculating cross sectional area. We do also agree about the lack of quality of imaging however we were unable to resolve the issue due to lack of modern ultrasound machines in the operating suite. |
| In general, I think there is a lot of superfluous information in the introduction that could be eliminated (such as the entire first paragraph). I would trim it down and just talk about why subclavian placement is done (advantages of this site over others) and then the purpose of your study.  here is complete unanimity regarding the value of central venous catheters in critically ill  patients. – I am not sure this statement is accurate and it is probably not needed. Consider modifying or deleting.  “Canulating the subclavian vein is part of everyday medical practice…” – I might be a relatively common procedure but I would not say it is part of everyday practice per se.  In terms of allowing for thrombotic events to be “abated” – perhaps it would be more accurate to say it lowers this risk as compared with the IJ or femoral sites  Rather than say the SC is preferred you might want to say “advantages of the SC site over the other options include…”  You might want to change the word fomenting to causing or something similar | We highly appreciate your input. Accordingly we have made some changes to the introduction to make it shorter and with simpler, less sophisticated vocabulary. |
| After 1 minute, the patients were safely placed in the Trendelenburg position. The  ultrasound probe would be placed parallel to the clavicle superiorly with slight acute angulation  towards the sternum, offering a coronal view – Could you include some images ? | Thank you so much.  We have added a photo of a patient showing the ultrasound probe position against the patient’s clavicle as described  The photo has been labelled as “fig 1” |
| it sounds like 1 sole operator performed all the procedures. Is that accurate? | That is correct. The most senior anesthesia consultant performed all measurements and venipunctures on the patients. That was done to minimize the limitations due to lack of way to standardize the procedure in terms of complication rates, mispunctures and ultrasound placement |
| How was the SC vein diameter measured? Short or long axis? At the point of maximal diameter? Can you show an image with measurement | Multiple measurement were taken and the greatest of them has been recorded in the database. An image has been attached in the supplementary file labelled as fig S1 |
| Figure 1 and 2 – please label x and y axis and please consider using different colors rather than different subtle shades of blue | Amendments to figure 2 and 3 ( previously labelled 1 and 2) have been made accordingly, thank you very much |
| What kind of ventilator was used? | GE Datex Ohmeda Avance S5 (Datex-Ohmeda, Ohmeda Drive, PO Box 7550, Madison, Wisconsin, 53707) |
| Figure 3 is not very clear. The images of the vein look somewhat different in each view and it isn’t clear to me that diameter is really different because it looks like the measurement was not taken at exactly the same spot? | We do agree, there are some variation in the images. We have noticed such variation in thin patients were chest expansion would greatly alter the ultrasound images. All the measurements would be recorded as the ultrasound is placed while the catheter is also being placed in the subclavian vein. |
| I think the main limitations concern how exactly the measurements were done, and possible lack of confidence that they were done the same way every time. It seems like it would be very easy to obtain different and conflicting results depending on how the measurements were done. | We are extremely grateful for your insightful comment.  We do agree there are some limitation to the methods used to measure the diameter of the subclavian vein however, we have made changes to the methodology while planning this study based on the limitation of previous studies to improve on the quality of evidence. Regardless, this warrants to need for further international collaborations and more prospective studies in favour of bettering patient safety and quality of care |
