# Supplementary material for: Lung deflation while placing a subclavian vein catheter: Our experience in minimizing the risk of pneumothorax
Source: PLoS One. 2023 Feb 2;18(2):e0281300. doi: 10.1371/journal.pone.0281300 (PMC9894405; doi:10.1371/journal.pone.0281300)
Supplement: Attachment — Submitted filename: Response to Reviewers.docx [file pone.0281300.s002.docx]

Prof. Emily Chenette

Editor-In-Chief

PLOS ONE

We are very grateful to you for providing us with the opportunity to submit a revised draft of our manuscript titled, “Title: Lung Deflation while Placing a Subclavian Vein Catheter: Our Experience in Minimizing the Risk of Pneumothorax” to Plos One. We are highly appreciative of the time and effort that have been dedicated to providing us with your valuable feedback on the manuscript. We are also thankful to the reviewers for their insightful comments. We have been able to incorporate changes to reflect most of the suggestions provided and have highlighted the changes accordingly in the manuscript.

Enclosed herewith is a point-by-point response to the reviewers’ comments and concerns.

| Comments | Reply |
| --- | --- |
| Reviewer #1: | |
| An excellent manuscript in its field, I have no corrections  I agree with the results | Thank you very much. The prospects of improving patient’s quality of care depend on repeated assessments of the measures taken. Therefore, this motivates us to explore unproven methods and validate the necessity to always question way to improve patients safety and outcome of care especially those who critically need it |
| Reviewer #2 |  |
| Considering the time-frame this work was performed (6 yrs), the numbers of devices placed was significantly low (n=123), averaging only 20.5 devices/year. This is considered under the minimal number to maintain a high standard of competency based upon published literature.  Was a powered study sample size calculated prior? A sample must be representative of the population, which this only included mechanically ventilated patients with selected inclusion criteria. How does this impact other patients who are critically ill or spontaneously breathing patients who may have a subclavian catheter placed also? Please consider this in your discussions.  Low sample sizes increase the margin of error into the analysis, potentially allowing for insufficient statistical power to answer the primary research question and creating a statistically non-significant result. The authors should clearly address this in the limitations sections of the manuscript. | Thank you very much for such an insightful comment.  We do agree and there are multiple factors that lead to the low patient numbers. The power indeed calculated during the initial phase of study design. In order for us to achieve 80% power with an alpha rate of 0.05 we needed 141 patients and our initial target was assigned to minimize the rate of type II error to its lowest rate possible.  However our initial study designed changed due to a significant difference in experience and complication rates per operator which would be a very important factor to eliminate as it is a major limitation to the validity of the anatomical significance this study provides. Having a single sole operator for the study limits the patient recruitment sample available for the study. To add, a significant number of patients have within our recruitment pool have not consented for the procedure and therefore the authors collectively agreed to analyze and publish the data with plans for multicentric international external validations |
| ABSTRACT - satisfactory.  Please define ‘catheter misplacement’ in the manuscript - it is also mentioned in the abstract as 0.8% and should be described as a’ primary malposition’ as this is determined during the insertion phase. Please see https://doi.org/10.5301/JVA.2011.8381 which describes primary and secondary malpositions/misplacements. | We highly appreciate your input.  The study protocol defined catheter malposition as a catheter that rests outsides the subclavian vein and the tip is not in its ideal position  The manuscript text has been edited accordingly as “misplacement” is an English technically vocabulary error. |
| KEYWORDS - please supply 6 keywords using MeSH terms whenever possible to improve searchability. Details can be found here - https://meshb.nlm.nih.gov/ | Thank you so much.  The Keywords have been double check and are infact mesh indexed |
| Please try and use third-person perspectives throughout the entire paper when presenting your research e.g. (the authors, this study, these findings, etc.) rather than first-person (we, our, us, etc.) - this makes for a more academically prepared manuscript. | We are highly thankful for your input and the text has been edited accordingly |
| a femoral catheter tip does not have its tip located in the SVC/IVC as it is not a centrally located device. It would be most likely in the iliac vein at best, depending on total catheter length and patients body habitus. I would avoid stating that femoral catheters are centrally placed UNLESS these devices are over 45-50cm in length, which most traditional CVC’s are not. Correct nomenclature and terminology is important and standardization should be considered when making reference to correct device locations.  Please see the following publications for further details - https://doi.org/10.1177/11297298221126818 | The manuscript text has been edited accordingly |
| I don’t think its necessary to describe the Seldinger technique - it is already well known. Consider removing | Thank you so much the manuscript has been edited accordingly |
| there is more recent evidence that supports the use of US guidance, with large number of systematic reviews - consider adding these, as Ref 4 is now a decade old  Suggested readings -  https://doi.org/10.2309/j.java.2019.004.002  https://doi.org/10.1016/j.jemermed.2020.07.039  https://doi.org/10.1007/s00134-019-05564-7  https://doi.org/10.1097/EJA.0000000000001383  <https://doi.org/10.1177/0885066619868164>  Canulating the subclavian vein is part of comm  on medical practice” - firstly, cannulating is spelt incorrectly, please correct.  Secondly, this is often not the case, as many trainees are most commonly taught the IJV approach first, which has been addressed in several publications. The subclavian approach has historically been a ‘high risk approach” however US has improved access to the vessel and actually increased the use of the Axillary vein, which has a clearer approach and visualization when using US. Consider this in your discussion also.  https://doi.org/10.1177/1129729819882602  https://doi.org/10.1007/s00134-019-05651-9  https://doi.org/10.1111/anae.15525  https://doi.org/10.1111/anae.15534  https://doi.org/10.1186/s13613-022-01065-x  https://doi.org/10.1186/s12871-021-01460-0  https://doi.org/10.7759/cureus.23823  https://doi.org/10.1177/11297298211038452  <https://doi.org/10.3390/diagnostics12010049>  REFERENCES  17/20 (85%) greater than 4 yrs old. Many of these papers describe practices that are no longer current in today's standards of practice. I would seriously consider updating many of these citations to align with current practices. I have listed a number of quality papers previously that you can consider with your revisions. | Thank you so much for your input and we highly appreciate your efforts  We do agree and we have made amendments to the manuscript text however  There are some articles that quite critical as the evidence regarding anatomical and procedural maneuvers are quite limited.  The references list has been updated and corrected accordingly as well. Thank |
| Please create a LIMITATIONS section - while this is addressed at the end of the discussion, it should have its own section. | Thank you so much and this has been done accordingly |
| FIGURES & TABLES  I would like to see some of the results tabulated for easy reading - currently, they are described in the results section, however the graphs do not do the results justice, making it harder to interpret. Please consider this in your future revisions.  Please ensure all figures have associated text to describe what is portrayed in the image.  Please provide a higher resolution image of Fig’s 2 & 3 - they are difficult to read and they also need a description associated with them. Please correct. | We highly value your input and therefore the figures have been amended and improved as well  Thank you |
