# Supplementary material for: Lung deflation while placing a subclavian vein catheter: Our experience in minimizing the risk of pneumothorax
Source: PLoS One. 2023 Feb 2;18(2):e0281300. doi: 10.1371/journal.pone.0281300 (PMC9894405; doi:10.1371/journal.pone.0281300)
Supplement: Attachment — Submitted filename: Response to Reviewers.docx [file pone.0281300.s003.docx]

Eyüp Serhat Çalık
Academic Editor
PLOS ONE

We are very grateful to you for providing us with the opportunity to submit a revised draft of our manuscript titled, “Title: Lung Deflation while Placing a Subclavian Vein Catheter: Our Experience in Minimizing the Risk of Pneumothorax” to Plos One. We are highly appreciative of the time and effort that have been dedicated to providing us with your valuable feedback on the manuscript. We are also thankful to the reviewers for their insightful comments. We have been able to incorporate changes to reflect most of the suggestions provided and have highlighted the changes accordingly in the manuscript.

Enclosed herewith is a point-by-point response to the reviewers’ comments and concerns.

| Editor/Reviewer Comment | Authors Response |
| --- | --- |
| Paragraph 1, 2nd and 3rd sentences of the introduction section should be reorganized (lines 65-68), after the change the meaning of the sentences is distorted. | Thank you so much for point the issue out and do acknowledge that it was unclear.  Alterations to sentences have been |
| You still have not defined the statistical methods you use in the statistics section (Chi-square test, Fisher's Exact Test, etc.). | We highly appreciate your input and we have described the statistical test used to compare the average means |
| The resolution of Figure 4 is still very bad, is there any chance to fix it? | Thank you so much.  We have reproduced the image and focused on minimizing the quality loss while collaging the pictures together. To ensure we provide the best quality we have also PACE ensure the picture meets the journal |
